# Variability in Water Capacity of Small-Leaved Linden Associated with Both the Presence of Honeydew and Various Sources of Pollution

**DOI:** 10.3390/plants12193443

**Published:** 2023-09-29

**Authors:** Agata Kwika, Anna Klamerus-Iwan, Anna Sadowska-Rociek

**Affiliations:** 1 Department of Ecological Engineering and Forest Hydrology, Faculty of Forestry, University of Agriculture in Krakow, Al. 29-Listopada 46, 31-425 Kraków, Poland; 2 Centre of Food Monitoring, Faculty of Food Technology, Malopolska University of Agriculture in Kraków, Ul. Balicka 122, 30-149 Kraków, Poland; anna.sadowska-rociek@urk.edu.pl

**Keywords:** urban forestry, fires, wettability, PAHs, petrogenic, pyrogenic

## Abstract

The process of water retention on the surface of the assimilation apparatus of plants is inextricably linked with the water capacity of tree crowns and depends mainly on the condition of the leaf itself. The main objective of the present study was to investigate how the honeydew coverage and the location of trees related to the content of polycyclic aromatic hydrocarbons affected the differences in the capability of small-leaved linden leaves to capture water. Honeydew coverage was determined with the use of AutoCAD, whereas the content of polycyclic aromatic hydrocarbons was determined using gas chromatography. The value of S (water capacity) was much lower before the appearance of honeydew on the leaf than at the peak moment of honeydew collection. This is due to the hydrophobic properties of the substance. It was also found that the content of polycyclic aromatic hydrocarbons (PAH) in leaves varied depending on the distance of trees from pollution sources, and it was found that the amount of PAHs increased with the growth of honeydew on leaves and in locations exposed to pollution. The highest S and the total amount of PAHs occurred with the combination of the largest amount of pyrogenic impurities with the highest amount of honeydew. Combing pollutants from the air by plants is a very important function, but it may also change the physical properties of leaves, such as wettability.

## 1. Introduction

The phenomenon of the penetration of precipitation through the tree canopy (throughfall) is determined by many ecological dependencies, and it tends to exhibit high temporal and spatial variability [1,2]. As a result of water retention on the surfaces of leaves, much less water reaches the forest floor [3]. Factors that may affect interception also include the amount of pollution, dust, or the hairs on the leaf surface [4]. The problem of interception has been the focus of hydrologists’ attention for many years [5,6,7,8], but recently it has been addressed to a greater extent in the context of global climate change and increasing air pollution, as well as the response of plants to these factors [9].

Developing industry and transport, progressing urbanisation, and increasingly frequent large-scale fires affect the entire ecosystem. They cause an upsurge in the amount of pollutants, deterioration of the air condition, and difficulties in the circulation of biogenic elements. A necessary solution must be to care for the maintenance and preservation of trees in good health, as natural air filters [10]. The leaves of both trees and shrubs are exposed to pollution, and they accumulate microdust to varying degrees [11]. In addition, particulate pollution (PM10 and PM2.5), tar substances, and oils rich in polycyclic aromatic hydrocarbons (PAHs), as well as metals deposited on the leaves can all cause changes to the hydrophilicity of plant material [12,13].

PAHs represent a group of organic compounds containing benzene rings in a linear, cluster, or angular arrangement [14], and they are formed in the process of the incomplete combustion of various substances, such as fuels, and also during fires. Many of these compounds show strong carcinogenic and mutagenic properties. On the other hand, the strong hydrophobic and lipophilic properties of most PAHs may be the reason for the easy accumulation of these compounds on the assimilation apparatus of plants [15], but also in the soil [16].

The amount of PAH sorption by plants depends to a large degree on such features of these plants as the surface or structure of leaves, and the thickness and chemical composition of the cuticle located on the surfaces of the assimilation apparatus. Corrugated leaves with a large surface show a much greater sorption of PAHs compared to small and smooth leaves [17]. Hydrocarbons associated with the fraction of waxes on the leaves are very difficult to remove; therefore, the dead parts of plants become an additional source of PAHs in the soil [17]. Large-area and rapidly occurring contamination of the assimilation apparatus arises in the vicinity of fires, because combustion causes substantial emissions of PAHs to the environment.

Honeydew may be another very important factor limiting the water capacity of leaves, as well as affecting the amount of pollutants retained. It appears periodically, because although it occurs naturally in the living environment of trees, it has a sticky structure. The chemical composition of honeydew is chiefly saccharides, which make up between 90% and 95% of the dry matter [18], and that favour water retention by the leaves.

Honeydew is an odourless and colourless sticky substance. Studies on its periodic occurrence and the amounts in which it appears had been conducted by Gałuszka and Tworek [19], who showed that the best conditions for the development of aphids, and thus for the abundant occurrence of honeydew, are those prevailing on sunny summer days with temperatures not exceeding 30 °C, whereas the one factor negatively affecting the formation of honeydew is long-term heavy rainfall.

Small-leaved linden (*Tilia cordata*) was selected for the analysis because it is common in most of Europe and Western Asia. In nutrient-rich habitats, it may enter the upper floor of the stand [20]. In stands composed mainly of light-demanding species, such as pine or pedunculate oak, small-leaved linden often occurs as a phytomeliorative admixture; it can also constitute a valuable ennobling component in beech stands [21]. The species in question forms tree stands in parks, suburban areas, in cities, and along the alleys in a moderate climatic zone [22]. Detailed research on the ability of small-leaved linden to collect pollutants has been conducted by Nawrot et al. [23]. The authors have shown that PM quantity on foliage increases over time, and that plants can be used as bio-monitors of air pollutants. In turn, also endorsed in the literature on the subject, we find methods for determining the content of hydrocarbons in plant material [24], and the degree of metal contamination therein [25,26].

We hypothesise that the amount of honeydew accumulated on the leaves of small-leaved linden, as well as leaf contamination by selected PAHs, both affect the amount of rainwater retained in the tree crowns.

The objective of the present study is to determine the factors affecting the water capacity of small-leaved linden (*Tilia cordata*) growing on research plots in three different zones. The zones differed in terms of the degree of pollution and the origin of that pollution. In addition to the location, the other aspect of our work is seasonality, which is associated with the presence of honeydew on the leaves. In particular, we set out to investigate whether there is a difference in the amount and in the diversity of PAHs retained on leaf blades depending on whether the contamination comes from ordinary road traffic or from a fire in a neighbouring stand.

In order to achieve the stated research objective, a series of laboratory tests was conducted, combining the simulation of rainfall on individual twigs with the measurement of the amount of retained water, depending on the degree of leaf coverage by honeydew and various sources of air pollution.

## 2. Results

**Figure 1:**
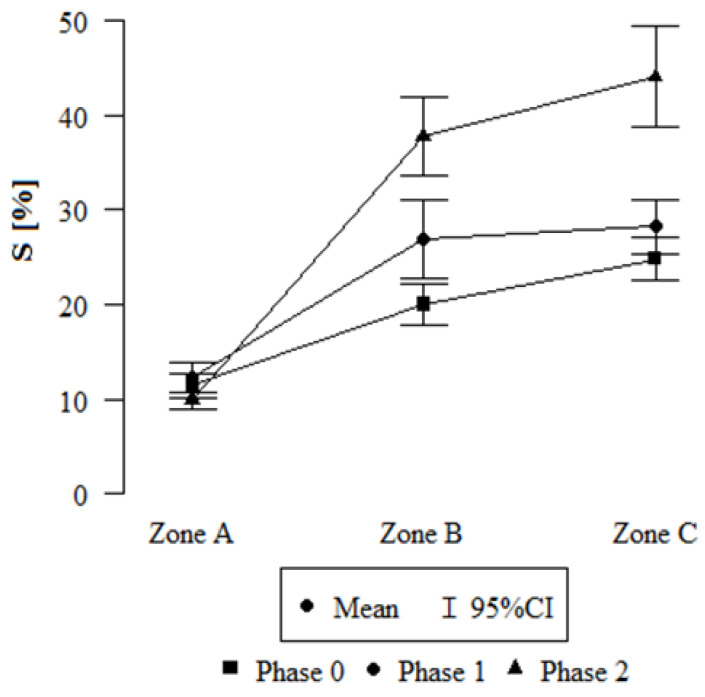
The value of S depending on the zone, for each phase; phase 0 (honeydew coverage up to 10% of the leaf surface), phase 1 (between 11 and 30%), and phase 2 (over 30% leaf coverage with honeydew); Zone A—rural zone, B—city centre, C—post-fire area.

The influence of the location and phase of honeydew on the water storage capacity S of small-leaved linden leaves is different for each of the three phases of sampling the assimilation apparatus (Figure 1), and it differs statistically significantly in relation to individual locations (Table 1). The lowest S, taking into account both the degree of leaf coverage with honeydew and the degree and type of contamination, occurs in phase 0 and location A. In location A, we also see the smallest differences between the phases in terms of S value. In locations B and C, the influence of the phase is visible in the variation of S (Figure 1).

Having applied linear regression, significant differences were found between the S of leaves at each location (A, B, and C). The highest mean S was found for leaves taken from zone C for phase 2. The regression parameter is 20.749; therefore, this combination of zone and phase additionally increases S by an average of 20.749 percentage points.

In zone B, the water capacity is, on average, 8.569 percentage points higher than in zone A, and in zone C, it is higher by 13.358 percentage points on average in relation to zone A.

### 2.1. General Dependence of S on SUM.PAH

**Figure 2:**
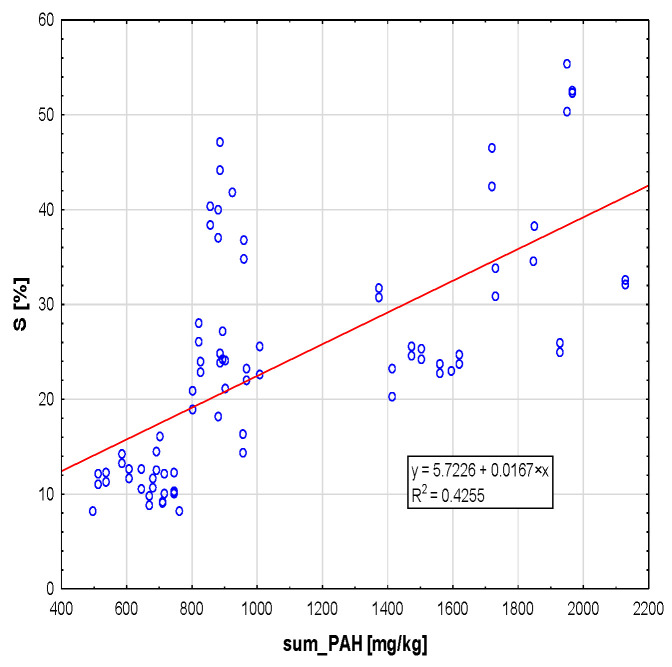
Correlation between SUM.PAH [mg/kg] and water storage capacity in tree crowns (S) for all phases and locations combined.

Spearman’s correlation coefficient for PAH and S is 0.752 (*p* < 0.001) for all locations and phases combined. As the sum of the PAH increases, the S also increases (Figure 2).

Linear regression with interactions showed that the phase, the location, and the interaction between the two have a significant influence on SUM.PAH. It was therefore important to study the same tree species in different phases and locations.

### 2.2. Comparison of Polycyclic Aromatic Hydrocarbons in Leaves Collected in Zones A, B, and C

**Figure 3:**
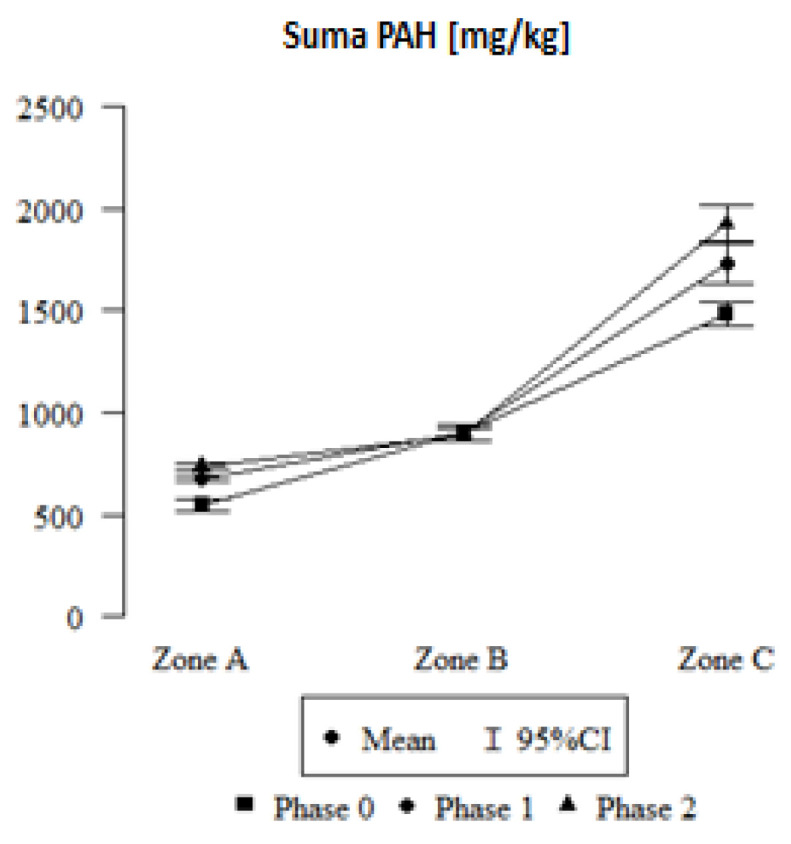
Total value of PAHs (SUM.PAH) in all phases and locations; phase 0 (honeydew coverage up to 10% of the leaf surface), phase 1 (between 11 and 30%), and phase 2 (over 30% leaf coverage with honeydew); Zone A—rural zone, B—city centre, C—post-fire area.

The amount of PAHs in small-leaved linden leaves varies depending on the location from which the assimilation apparatus was collected, and the phase in which it was collected. Linear regression showed that S correlates with the concentration levels of SUM.PAH, which is the sum of concentrations of the following compounds: NP—naphthalene; MeNap2—2-methylnaphthalene; MeNap1—1-methylnaphthalene; ACE—acenaphthene; ACY—acenaphthylene; FL—fluorene; PHE—phenanthrene; ANT—anthracene; FLA—fluoranthene; PYR—pyrene; BaA—benzo[a]anthracene; CHR-chrysene; BbF-benzo[b]fluoranthene; BkF-benzo[k]fluoranthene (Figure 3).

In zone B, the regression parameter is 351.07; therefore, it raises the SUM.PAH by an average of 351.07 µg/kg compared to zone A, while in zone C, the SUM.PAH is on average 935.566 µg/kg higher than zone A. When comparing SUM.PAH in individual phases, phase 1 raises SUM.PAH by an average of 130.118 µg/kg, and phase 2 raises it by 187.866 µg/kg compared to phase 0 (Table 2).

In the case of zone B interaction with phase 1, we observe that the regression parameter is −130.782; therefore, this combination of zone and phase additionally reduces SUM.PAH by an average of −130.782 µg/kg. A decrease by −192.443 µg/kg is also observed when analysing the interaction of zone B with phase 2.

In the case of zone C interaction with phase 1, the regression parameter is 112.588; therefore, this combination of zone and phase additionally raises SUM.PAH by an average of 112.588 µg/kg. Leaves taken from zone C in phase 2 retained more SUM.PAH by an average of 252.02 µg/kg.

**Figure 4:**
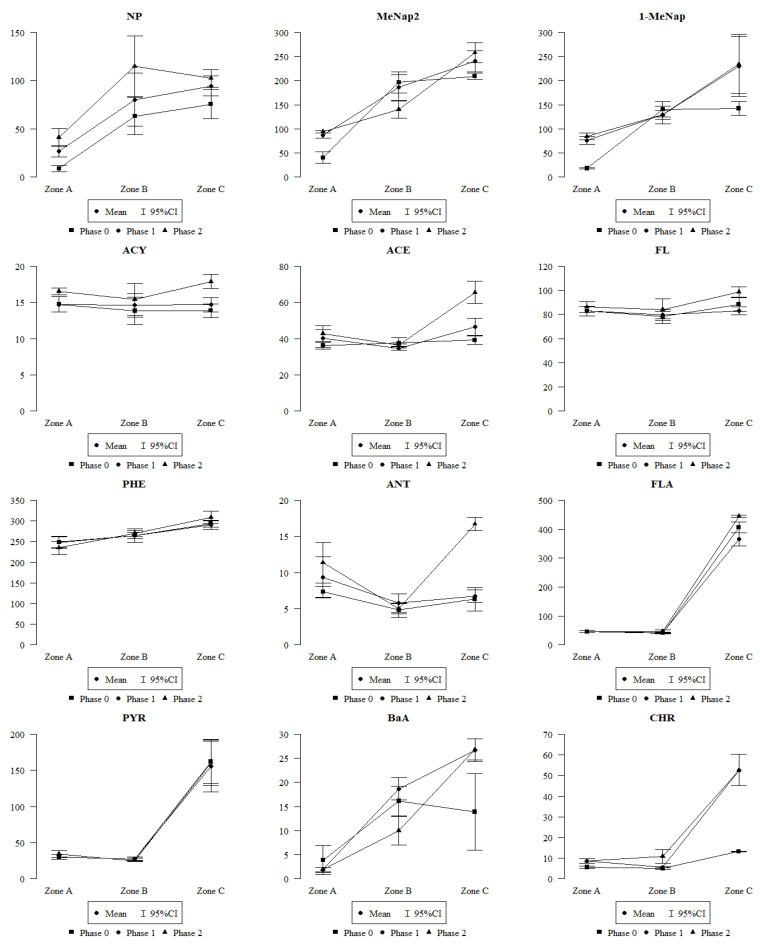

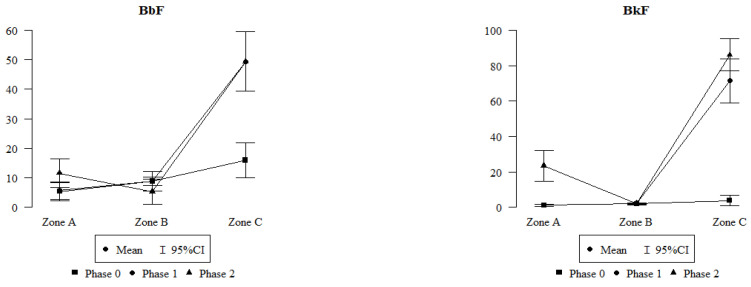
The impact of the pollutants [µg/kg] on the species and locations; Zone A—rural zone, B—city centre, C—post-fire area; NP—naphthalene; MeNap2—2methylnaphthalene; MeNap1—methylnaphthalene; ACE—acenaphthene; ACY—acenaphthylene; FL—fluorene; PHE—phenanthrene; ANT—anthracene; FLA—fluoranthene; PYR—pyrene; BaA—benzo[a]anthracene; CHR—chrysene; BbF—benzo[b]fluoranthene; BkF—benzo[k]fluoranthene.

Individual PAHs do not occur separately. Therefore, the image in Figure 3 is significant; however, an analysis of individual aromatic hydrocarbons linked the source of contamination to the sampling location (Figure 4).

## 3. Discussion

The process of water retention on the surface of the assimilation apparatus of plants is closely related to the water capacity of crowns, measured by field and laboratory tests [1]. Interception depends for the most part on the condition of the assimilation apparatus [6]. The process of interception in leaves of trees and shrubs is also influenced by other factors, such as, for instance, their long exposure to pollution, resulting in a change in the texture of the assimilation apparatus [27]. However, they differ in terms of the amount of pollutants that they can retain on their surface [28]; therefore, it is reasonable to examine the leaves in terms of contamination both in areas of high exposure to pollution as well as in areas seemingly not exposed to large amounts of pollution. From the present work, it can be concluded that the increase in the amount of pollutants on the surface of the assimilation apparatus hinders the process of water interception by the plant.

Based on the graphs (Figure 3 and Figure 4) representing the amount of individual PAHs in small-leaved linden leaves for phase 0, 1, and 2 in each of the three zones, it can be concluded that the dominant hydrocarbon in each location, regardless of the degree of leaf coverage by honeydew, is Phenathrene, i.e., a hydrocarbon obtained from coal tar, and in the natural environment its presence is most often the result of hard coal combustion. Its highest level existing in Cracow (zone 3) is related to the on-going forest fire. Remaining at a slightly lower level is the concentration of 2-methylnaphthalene [C11H10], which is a compound formed as a result of incomplete combustion of organic compounds, and whose presence is also found in engine exhaust, tar or cigarette smoke. Its highest level, recorded in Cracow (zone 2), is due to the increased vehicle traffic. Fluorene, whose amounts are almost equal in each location and phase, is formed—like many other PAHs—during the incomplete combustion of diesel fuels (Figure 4). The calculated diagnostic ratios [29] BaA/(BaA + CHR) and FLA/(FLA + PYR) (https://data.mendeley.com/datasets/xt63zr4f8n/1 (accessed on 29 August 2023)) clearly indicate the separation between petrogenic and the coal combustion origin of the PAHs. Diagnostic ratios were calculated to determine the sources of PAHs [29]. The presented results also show high values for some PAHs in the leaves of linden trees growing in rural areas, away from the city, from industrial plants, or from highways. However, in each case, the PAH values for the 0 phase—that is, before the appearance of honeydew on the leaves—were lower than in the subsequent phases, and this confirms the hypothesis that honeydew as a viscous substance rich in saccharides favours the retention of harmful substances on the surface of the leaves and hinders their neutralisation.

The analyses that we conducted almost unambiguously lead to the conclusion that the location of trees and the honeydew coverage of the assimilation apparatus combined play a key role in the process of water retention by the small-leaved linden. Our research indicates that pollutants accumulated after a fire and PAHs accumulated in leaves can affect the amount of precipitation retained. Furthermore, plants that have been weakened by pollution are often more susceptible to bacterial and fungal infections [12,30], which also modifies the surface of the leaf, and thus its ability to retain water.

In the first phase, i.e., before the appearance of honeydew on the leaf blades, more water flowed down the leaves, and the S of the assimilation apparatus was lower. However, with the increase in the amount of honeydew on the leaves, which due to its sticky structure affects the retention of pollutants, more water was retained on the surfaces of the leaf blades, although, unfortunately, the plant does not use that retained water. This is due to the hydrophobic properties of honeydew. Changes in the value of S occur both in heavily polluted areas, such as the city of Cracow (B) or zone C, located in Olkusz in the area after the previous occurrence of a forest stand fire, and in areas with a much lower amount of atmospheric pollution, such as a town located in the forest cover of a rural area (Figure 2 and Figure 3). By performing a series of measurements at time intervals for two urban locations and one rural location, we can formulate conclusions as to the mechanism that caused the effect of changes in leaf hydrophilicity depending on the amount of honeydew and the potential amount of pollutants. The results presented in this paper are consistent with the results of research conducted by Schreuder et al. [31], who demonstrated that plants covered with waxes in less polluted areas retain water to a lesser extent. In terms of seasonal changes and the amount of pollutants, they are comparable to the results presented by Klamerus-Iwan A. et al. [32].

Pollutants in various states of aggregation—solid, gaseous or liquid—are substances that have a negative impact not only on the environment and the growth and vegetation of plants, but also on human and animal health [33,34]. In turn, the impact of microdusts on health depends on their chemical composition and exposure time [35,36]; unfortunately, they are harmful because they include carcinogenic PAHs and heavy metals [37,38,39]. These compounds, which come from fires, as well as from oil and coal combustion, take the form of gases, aerosols, and dusts. They are a derivative of the transport and energy industries, and they can be classified under the category of anthropogenic pollutants [40]. With the current concentration in the atmosphere, the negative impact of PAHs on vegetation is small [41]. However, the urban environment is fundamentally different from other known biotopes, because it has been transformed and adapted to human requirements, and as a result, the proportions between abiotic and biotic components of the environment have changed [42]. The results of the analyses conducted for the purpose of the present work are surprising, because they indicate that the presence of PAHs is not only the feature of urban areas—which is illustrated with the examples of Cracow and the post-fire area in Olkusz; instead, the high level of PAHs was also recorded in rural areas, seemingly distant from the sources of pollution (Figure 2). The location related to the degree of pollution gave similar results for three coniferous species [43] by changing the increase in S in the most contaminated place.

Waxes of different chemical composition and structure found on the surface of the leaf blade have different viscosity; they favour the adhesion of microdust to their surface, and some of the microdust also penetrates into the wax layer [44,45]. The level of particulate matter on plant foliage also depends on their distance from the source of emission [46]. The main path of accumulation and retention of PAHs in plant organisms is from the air to the leaf surface, while Kipopoulou et al. [47] in their studies demonstrated that the concentrations of polycyclic aromatic hydrocarbons in plant tissue are similar to those found in urban air. In Cracow, the level of airborne pollutants is one of the highest in Europe; this is due to its geographical location, the main directions of wind, and the current deposition of industrial dust [48,49]. Whereas more and more work is being conducted on urban air pollution, the additional impact of fire pollution on the water capacity of tree crowns has yet to be explored.

The present study allows us to conclude that with the increase in pollution (in the case of Zone C related to the fire) and with the most intense appearance of honeydew, S takes the highest values, and, therefore, the least water penetrates through the assimilation apparatus and flows down the surface of the leaves to the lower layers of the ecosystem.

This is probably due to the fact that honeydew is a sticky substance, in combination with the fact that impurities and dust accumulated on the surface of the leaves clog the stomata and naturally hinder the absorption of water, and only contribute to its retention and re-evaporation from the surface of the leaves.

Different species accumulate harmful substances to varying degrees. Detailed research on the ability of small-leaved linden to collect pollutants was conducted by Nawrot et al. [23]. In turn, based on research conducted in Polish cities on the small-leaved linden, Popek et al. [50] demonstrated its ability to accumulate pollutants such as dust, PAHs or heavy metals in the leaves, which affects the potential improvement of air quality in the vicinity of this tree species in the urban environment. Confirmation of this fact is also reflected in our results (Figure 3 and Figure 4), where in different locations, due to the amount of pollutants, we obtained different concentrations of PAHs in the leaves. The results of our research turned out to be in line with the universally acknowledged view that vegetation in cities plays a particularly important role in cleansing the atmosphere.

An important observation is also the fact that the water capacity of tree crowns is not a constant value, and, furthermore, the factors that change it cannot be considered separately. Pollution, diseases, and natural seasonal changes in this honeydew can all strengthen or weaken each other.

## 4. Materials and Methods

### 4.1. Sampling

**Figure 5:**
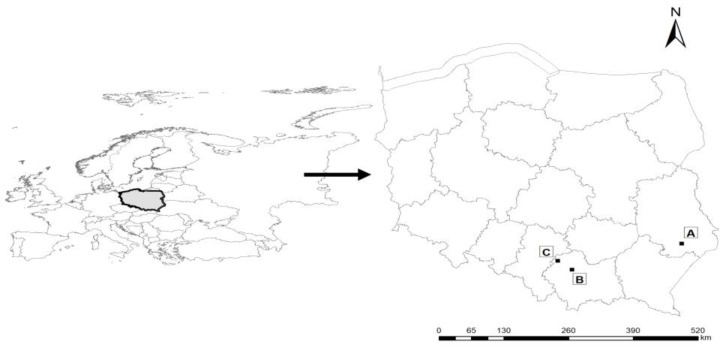
Location of measurement points A, B, and C.

Samples for testing were collected in three locations. The first (A) was situated in mid-field woodlots of Roztocze Środkowe region (rural zone) in Bondyrz [50.58425, 23.07860], far away from highways, buildings, or industrial centres. Two further surfaces were situated in places subjected to different sources of air pollution. In the second location (B) [50.08283, 19.95298] (Kraków), the impact of pollution, originating mainly from car traffic, was recorded, whereas in the third location (C) [50.27497, 19.55616] (Olkusz), the impact of pollution from fires was documented (Figure 5).

In each location, samples of leaves for testing were collected in three repetitions, differing in terms of the presence, and intensity of the presence, of linden honeydew; these were called phases. Phase 0 was collected at the beginning of June 2021, i.e., before the most intense occurrence of small-leaved linden honeydew; phase 1 was collected at the beginning of July 2021, at the time of its most intense occurrence; and phase 2 was collected at the beginning of August 2021, i.e., at the end of honeydew occurrence on leaves. When collecting samples, the criteria necessary for collecting plant material were taken into account. The air temperature during the collection of the assimilation apparatus for each phase of honeydew coverage did not exceed 30 °C, and no heavy rainfall was recorded, in accordance with the recommendations of Gałuszka and Tworek [19].

Within each of the three zones, trees of the *Tilia cordata* species were identified on the study plots, from which 20 branches, approximately 0.5 m long, were collected. Trees around 20–40 years old with a properly developed crown were selected. Leafy branches were collected with shears on a telescopic boom from different heights and places within the crown.

### 4.2. Determination of the Size of Water Capacity

Water capacity (S) of the leaves was treated as the difference between the amount of water used for rainfall simulation and the amount of water that was not left on the twigs. Distilled water was used to simulate the rainfall—that is, water free of impurities and ions—in order to obtain an equally clear image of the impact of honeydew, and of impurities, on each simulation.

Rainfall over the branches was artificially simulated in the laboratory [11]. Selected twigs, on which the degree of honeydew coverage was determined, were simulated with a constant dose of precipitation (P). P was measured using a calibrated sprinkler and then reweighed when wet. The total P was obtained from the previous calibration, amounting to 100 g. The twigs were sprayed from a constant distance of 0.4 m, and their orientation during spraying was similar to the natural arrangement when growing on a tree.

The analyses were carried out in five replicates, for each location, in three phases. Tests were carried out indoors, with no wind, with controlled humidity (48%), at the temperature of 21 °C. Temperature is important for the density of the water, and the adhesion of the droplets to the plant material.

### 4.3. Determination of the Degree of Leaf Coverage by Honeydew

**Figure 6:**
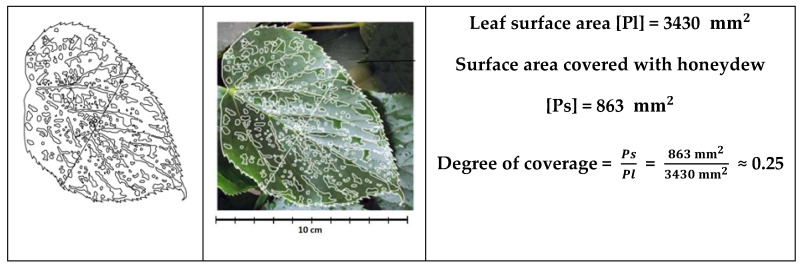
Leaf sample with the method of measuring the degree of honeydew coverage—phase 1.

Single leaves taken from larger twigs were used to determine the extent of their surface coverage by honeydew for each phase of appearance of the latter. Then they were photographed, scaled, and measured using AutoCAD software. The outline of the leaf surface and outlines of honeydew drops were made depending on the phase and intensity of the occurrence of the latter. Ultimately, the coverage was defined as the ratio of the degree of coverage by honeydew to the size of the leaves (Figure 6).

The degree of leaf coverage with honeydew was assigned to the following phases: phase 0 (up to 10% of the leaf surface), phase 1 (between 11 and 30%), and phase 2 (over 30% of leaf cover with honeydew).

### 4.4. Measurement of the Content of Selected PAHs in Leaf Samples

From each sample of twigs, leaves were collected, dried, and ground in a mortar in order to prepare them for chemical analysis. In the laboratory, the elements of the small-leaved linden assimilation apparatus were measured for the content of polycyclic aromatic hydrocarbons (PAHs). PAHs’ concentration levels in the assimilation apparatus were obtained, depending on the location and time of material collection for analysis. Sample preparation procedure was based on the previous reports [30]. The method was based on modified QuEChERS concept that was optimised for plant origin samples and in-house validated [51]. All values met the criteria set by Regulation (EC) No 333/2007 (amended on 1 January 2023). (https://eur-lex.europa.eu/legal-content/en/ALL/?uri=CELEX%3A32007R0333; accessed on 15 May 2023).

Analyses were performed on a Varian 4000 gas chromatograph coupled with a mass spectrometer (GC/MS) (Agilent Technologies, Santa Clara, CA, USA) featuring a CP-8410 auto-injector (Bruker, Billerica, MA, USA) with a DB-5MS column (30 m × 0.25 mm) × 0.25 µm; Agilent Technologies, Santa Clara, CA, USA). The injector temperature was set to 270 °C and the injection volume to 1.0 µL. Data acquisition and processing was performed using the Varian Start Workstation software (Agilent Technologies, Santa Clara, CA, USA) and the NIST 2.0 library (National Institute of Standards and Technology, Gaithersburg, MD, USA). The following PAHs’ levels have been established: NP—naphthalene; MeNap2—2methylnaphthalene; MeNap1—1-methylnaphthalene; ACE—acenaphthene; ACY—acenaphthylene; FL—fluorene; PHE—phenanthrene; ANT—anthracene; FLA—fluoranthene; PYR—pyrene; BaA—benzo[a]anthracene; CHR—chrysene; BbF—benzo[b]fluoranthene; BkF—benzo[k]fluoranthene; B[a]F—benzo[a]fluoranthene; B[e]P—benzo[e]pyrene; B[a]P—benzo[a]pyrene; I[cd]P—indeno[1,2,3-c,d]pyrene; D[ah]A—dibenzo[a,h]anthracene; B[ghi]P—benzo[g,h,i]perylene. Based on the obtained values for individual PAHS, the diagnostic ratios [29] BAA/(BAA + CHR) and FLA/(FLA + PYR) was calculated, which can additionally confirm the source of the origin of pollution.

### 4.5. Statistical Analysis

Correlations between quantitative variables were analysed using Spearman’s correlation coefficient. The impact of individual variables on S and SUM.PAH was examined by applying multiple linear regression. The advantage of using linear correlation analyses is the ease of interpretation of the identified dependencies. The regression used reveals a significant, positive dependence of S on SUM.PAH; therefore, the higher the SUM.PAH, the higher the S. In addition, significant changes in S were observed depending on the zone and the phase of the measurements. The analysis of the impact of phase and zone on quantitative variables was performed using the linear regression method with the interaction of phase and zone. The results have been presented as regression model parameter values with a 95% confidence interval. The analysis adopted a significance level of 0.05. Thus, all *p* values below 0.05 were interpreted as significant correlations. The analysis was performed using the R software, version 4.3.0 [52].

## 5. Conclusions

In the study here presented, various factors affecting the water capacity of small-leaved linden were observed, including the location of trees, the degree of leaf cover with honeydew, and the time in which the material for testing was collected. The following conclusions were formulated:

1. Significant differences in the S of small-leaved linden leaves in each of the three locations were recorded, related to the quantity and quality of PAHs in the leaves;
2. Differences in the amounts of PAHs in leaves were observed, depending on the emission sources (vehicle traffic and fire), as well as an increase in the amount of PAHs with an increase in the amount of honeydew on the leaves;
3. Differences in the ability to retain water were observed with a large amount of honeydew on the surface of the assimilation apparatus.

Research on the impact of fire pollution on plants and their retention properties is very insufficient. New York in 2023, after the fires in Canada, recorded 140 µg/m^3^ PM10 in the air, which was well above the standard of 50 µg/m^3^. Many cities around the world, even without fires, have average pollution levels that exceed the standard pollution concentrations, and fires that are becoming more and more frequent further increase these values. These data clearly indicate that more research is needed on the impact of fire effects on the retention properties of forest ecosystem elements.

## Figures and Tables

**Table 1:** Linear regression results showing the influence of location and phase on S; phase 0 (honeydew coverage up to 10% of the leaf surface), phase 1 (between 11 and 30%), and phase 2 (over 30% leaf coverage with honeydew); Zone A—rural zone, B—city centre, C—post-fire area.

Variable	Value	Parameter	95%CI		*p*
Zone	Zone A	ref.			
	Zone B	8.569	4.141	12.997	*p* < 0.001 *
	Zone C	13.358	8.93	17.786	*p* < 0.001 *
Phase	Phase 0	ref.			
	Phase 1	0.828	−3.6	5.256	*p* = 0.711
	Phase 2	−1.522	−5.95	2.906	*p* = 0.496
Interactions	Zone B: Phase 1	6.139	−0.124	12.402	*p* = 0.055
	Zone C: Phase 1	2.59	−3.673	8.853	*p* = 0.413
	Zone B: Phase 2	19.231	12.968	25.494	*p* < 0.001 *
	Zone C: Phase 2	20.749	14.486	27.012	*p* < 0.001 *

*—statistically significant differences; Cl—confidence interval; parameter—percentage points of water capacity.

**Table 2:** Statistical correlations of mutual interactions between the zone, the phase, and the SUM.PAH; Zone A—rural zone; B—city centre; C—post-fire area; phase 0 (honeydew coverage up to 10% of the leaf surface), phase 1 (between 11 and 30%), and phase 2 (over 30% leaf coverage with honeydew).

Variable	Value	Parameter	95%CI		*p*
Zone	Zone A	ref.			
	Zone B	351.07	274.126	428.014	*p* < 0.001 *
	Zone C	935.566	858.621	1012.51	*p* < 0.001 *
Phase	Phase 0	ref.			
	Phase 1	130.118	53.173	207.062	*p* = 0.001 *
	Phase 2	187.866	110.921	264.81	*p* < 0.001 *
Interactions	Zone B: Phase 1	−130.782	−239.597	−21.966	*p* = 0.019 *
	Zone C: Phase 1	112.588	3.773	221.404	*p* = 0.043 *
	Zone B: Phase 2	−192.443	−301.259	−83.628	*p* = 0.001 *
	Zone C: Phase 2	252.02	143.205	360.836	*p* < 0.001 *

*—statistically significant differences; Cl—confidence interval; parameter—SUM.PAH [µg/kg].

## Data Availability

https://data.mendeley.com/datasets/xt63zr4f8n/1 (accessed on 29 August 2023).
